# Fracture Resistance and Initial Penetration Time of a Novel Zirconia Crown Design for Simplifying Future Endodontic Treatment: An In Vitro Study

**DOI:** 10.3390/dj12120385

**Published:** 2024-11-26

**Authors:** Mohammed Mashyakhy, Hafiz Ahmed Adawi

**Affiliations:** 1 Department of Restorative Dental Science, College of Dentistry, Jazan University, Jazan 45142, Saudi Arabia; 2 Department of Prosthetic Dental Science, College of Dentistry, Jazan University, Jazan 45142, Saudi Arabia; haadawi@jazanu.edu.sa

**Keywords:** zirconia crown, novel design, fracture resistance, failure mode, access opening

## Abstract

**Objectives**: This study aimed to investigate the fracture strength of a novel-designed Zirconia crown before and after access opening, and to evaluate the mode of fracture and the time needed for initial penetration through the crown. **Methods**: This study involved the design and testing of 60 zirconia crowns, divided into three groups (20 crowns each) to compare different structural designs. Group 1 (Control) used a conventional full zirconia crown. Group 2 (Novel Design) featured a zirconia crown with an impermeable ceramic-filled opening. Group 3 (Modified Novel Design) included a zirconia crown with a permeable composite-filled opening. Each crown was designed using CAD/CAM technology with digital and cone beam CT scans to locate the pulp chamber accurately. The crowns were tested in two experiments. Experiment (A): Ten crowns from each group underwent a fracture test. Experiment (B): Ten crowns per group underwent an access cavity penetration followed by a fracture test. Key variables assessed included fracture strength, penetration time, and failure mode for each crown design, both before and after access opening. Data were analyzed using SPSS, with a significance threshold of *p* < 0.05. **Results**: The highest value of fracture strength before initial penetration was recorded for zirconia porcelain crowns (760.2 ± 25.2 MPa), while the lowest value was recorded for zirconia composite crowns (652.4 ± 25.9 MPa). The least time for initial penetration was recorded for zirconia composite crowns (2.5 ± 0.8 s). The difference in failure mode among the crowns was significant (*p* < 0.05) before initial penetration. All zirconia composite crowns showed crown fracture and core cracked, while all full zirconia crowns showed crown fracture only. The difference in failure mode before and after penetration was only significant for zirconia composite crowns. **Conclusions**: The modified novel-design crown (zirconia composite) could be an excellent choice when placing new prosthesis, since the crown provides easy access and a predictable guide to the root canal system and has good resistance to fracture before and after performing root canal therapy (RCT).

## 1. Introduction

It has been observed that the survival rates for pulp vitality are approximately 84.4% for single-crowned teeth and 70.8% for abutment teeth after 10 years. These rates decline to 81.2% and 66.2%, respectively, after 15 years [1]. Not surprisingly, teeth with a history of multiple restorations have a higher risk of developing endodontic disease within three years of crown cementation [2]. Since many teeth may require root canal treatment (RCT) or re-treatment over time after being crowned [1,3,4,5], accurate penetration through the crown to the dental pulp is essential for successful RCT [6]. However, creating an access cavity through an existing prosthesis presents a known challenge [7,8]. A recent in vitro study proposed a novel design for conventional zirconia crowns that includes a non-permeable hole, filled with a ceramic of a different shade, positioned directly above the pulp chamber of the crowned tooth [8]. The results of this preliminary study were promising. The time required for access cavity preparation through the novel crown was shorter, and the overall quality of the access was within an acceptable range compared to controls.

Partially stabilized zirconia represents a new generation of dental materials, characterized by high fracture toughness, strength, and translucency. Its durability and excellent wear resistance make it particularly suitable for monolithic, fully anatomic, and highly aesthetic prostheses, such as posterior full crowns and short-span fixed partial dentures. Additionally, the tooth preparation required for monolithic zirconia crowns is conservative, typically ranging between 0.5 mm and 1 mm. The adhesion and bonding of zirconia to dental resins have been extensively studied. Surface treatments like sandblasting, combined with the application of specific primers, have been shown to significantly enhance the stability and retention of zirconia crowns [9,10,11,12].

As a continuation of testing this novel crown design, a modification was introduced. This study aimed to investigate the strength of both the novel crown and its modification before and after access opening, as well as evaluate the mode of fracture. The time required for initial penetration through the crowns was also measured. The null hypothesis was that there are no differences between the novel crown design, its modification, and conventional full zirconia crowns in terms of compressive strength before and after access opening, initial penetration time, and mode of fracture.

## 2. Materials and Methods

**Figure 1:**
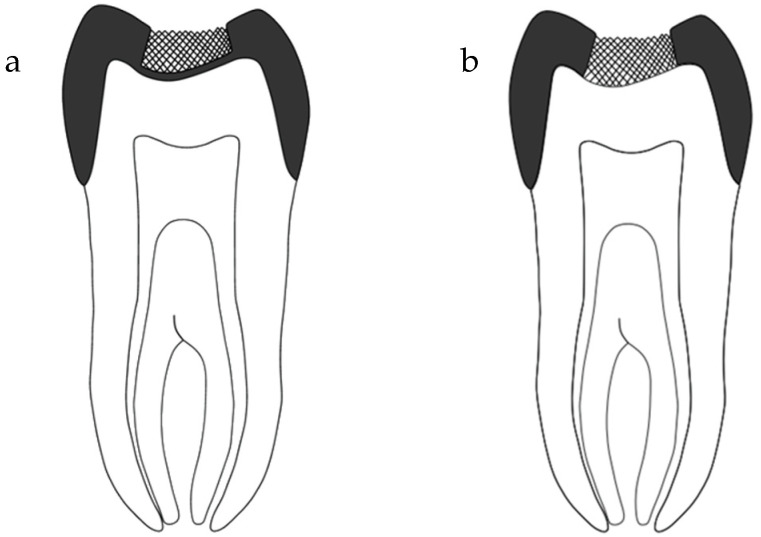
A schematic representation of (**a**) the novel design and (**b**) the modified novel design.

Before commencing the study, ethical approval was obtained from the Ethical Committee at College of Dentistry, Jazan University (Reference No. CODJU-197512). The fabrication method for the novel crown design is described in detail in a previous study [8]. In brief, the novel crown is created by accurately locating the pulp chamber using cone beam computed tomography (CBCT). A full-coverage zirconia crown is then designed and milled using a computer-aided design/computer-aided manufacturing (CAD/CAM) system, with a 3 mm-radius impermeable hole positioned over the identified pulp chamber. The hole is cut occlusally, leaving a base thickness of approximately 0.5 mm. The occlusal hole is filled with ceramic in a different shade, serving as a guide for access through the crown. A schematic representation of the novel design and its modification is shown in Figure 1a,b.

**Figure 2:**
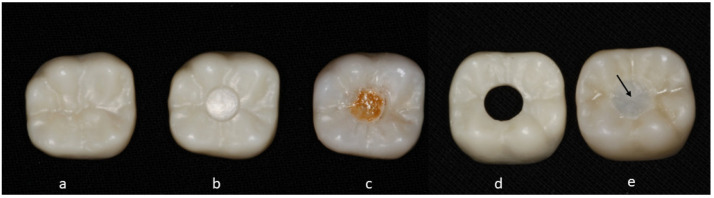
The experimental groups: (**a**) conventional zirconia crown, (**b**,**c**) zirconia crown with an impermeable hole filled with ceramic, and (**d**,**e**) zirconia crown with a permeable hole filled with composite (black arrow).

For the current experiment, a total of 60 crowns (n = 20 per group) were designed and divided into three groups: Group 1 (Control—Full Zirconia): Conventional zirconia crown, partially stabilized, translucent, and multilayers zirconia 5 (UPCERA ST-ML, Guangdong, China) was used in the study (Figure 2a); Group 2 (Novel Design—Zirconia Ceramic): zirconia crown with an impermeable hole filled with ceramic (VITA VM, VITA, Bad Säckingen, Germany) (Figure 2b,c); and Group 3 (Modified Novel Design—Zirconia Composite): zirconia crown with a permeable hole filled with composite (Figure 2d,e). The modified novel design was fabricated similarly to the novel design, except the hole was fully permeable (through and through) and filled with composite (Z100, 3M ESPE, St. Paul, MN, USA) of a different shade.

The fabrication requests were sent to the milling machine to create the three types of zirconia crowns, using zirconia block materials from the same STL file. The crowns were then sintered in a furnace at 1350 °C for 120 min (VITA Vacumat V60 I-line ceramic Furnace; VITA, Germany). For the Zirconia Ceramic group, the hole was filled with stained feldspathic ceramic (VITA, Germany) and sintered again in the furnace at 1350 °C for 120 min. The Zirconia Composite group underwent airborne-particle abrasion using silica-modified Al2O3 particles (50 μm) for 30 s at 1 MPa. Afterward, the crowns were cleaned with 95% ethanol and air-dried to remove any sand particle remnants. Z-Prime Plus (Bisco, Schaumburg, IL, USA) zirconia primer was applied to the hole’s border using a micro-brush and dried with an air spray for 3–5 s. The surface was then bonded with Porcelain Bonding Resin (Bisco) and light-cured for 40 s using a curing light (Kerr, Orange, CA, USA). The hole was sealed with tin foil on the intaglio surface of the crown, filled with Z100 composite (3M ESPE, St. Paul, MN, USA), and light-cured for 40 s using the same curing light.

The experiment was conducted in three phases:

(1) Experiment (1) Fracture test: Ten crowns from each group were subjected to a fracture test without any prior preparation (no access cavity; control group).
(2) Experiment (2) Cavity perforation time: The remaining ten crowns from each group were subjected to access cavity penetration, and the time required to penetrate through the different materials was measured.
(3) Experiment (3) Fracture test of the accessed crowns: The crowns from the test groups, after undergoing access cavity preparation, were then subjected to a fracture test.

**Figure 3:**
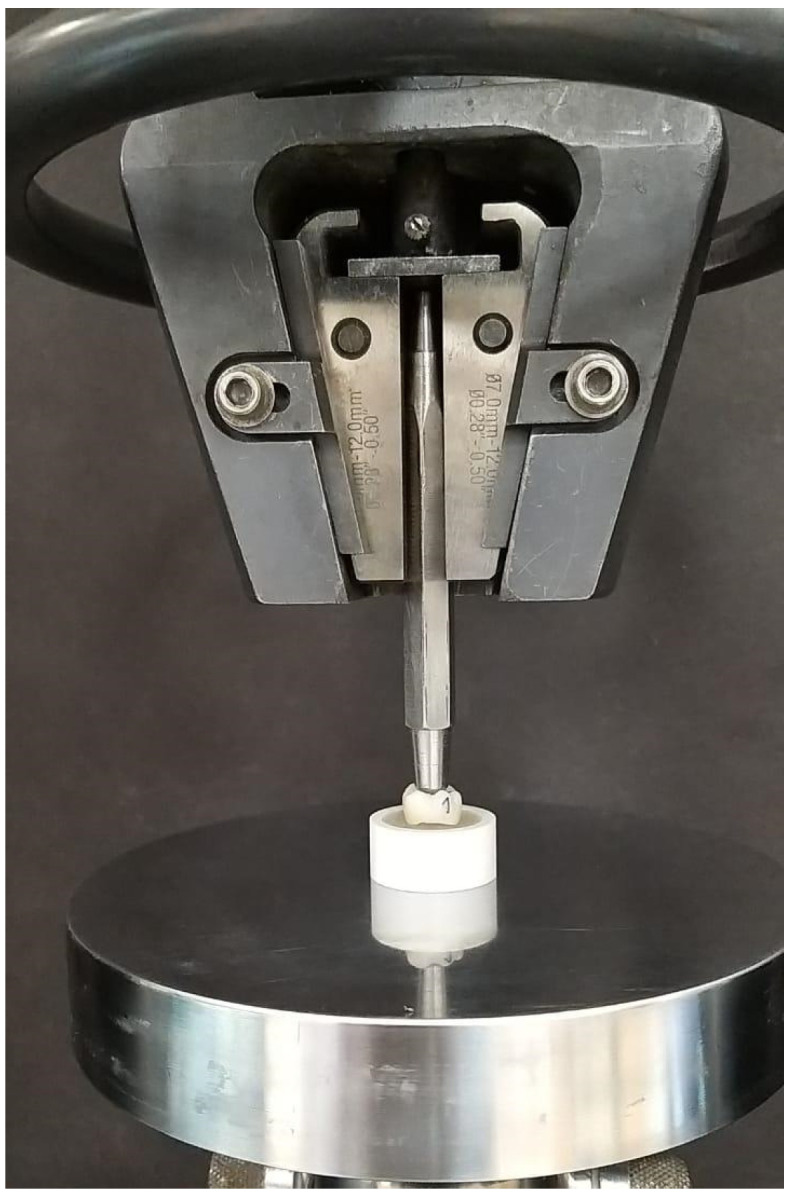
Fracture test using universal testing machine.

In Experiment 1, the crowns (n = 10 per group) were filled with auto-polymerizing acrylic resin (Caulk Orthodontic Resin; Dentsply Caulk, York, PA, USA) and stabilized in a stainless-steel holder attached to a universal testing machine (Model 4481, Instron Corp., Norwood, MA, USA) using an auto-polymerizing acrylic block (Figure 3). Pressure was applied through a clear polyethylene sheet (thickness: 0.04 mm) covering the occlusal surface of the crown, using a 5 mm-radius stainless steel bar with a flat circle diameter of 2.5 mm at the tip. The contact surface area was approximately 4.9 mm^2^. The load applied to the occlusal surface was aligned parallel to the crown axis at a crosshead speed of 0.5 mm/min until the crown failed. The load at fracture was recorded, and the compressive strength was calculated using the following equation: CS = F/A, where CS is the compressive strength, F is the force or load at the point of failure, and A is the initial cross-sectional surface area.

In Experiment 2, one operator, who had no prior experience with access openings through crowns, performed the initial penetration for all three groups (n = 10 each) using a 1.8 mm coarse round diamond bur (TIA DIAMOND, Tia Dent Inc., New Jersey, USA). A new bur was used for each crown. The time required for initial penetration (the duration in seconds from the start until the bur completely penetrated the crown) was recorded using a stopwatch. Three samples were excluded from the analysis in this experiment due to a miscalculation of time; however, they were included in the subsequent test.

In Experiment 3, the crowns (n = 10 per group) that underwent access cavity penetration in Experiment 2 were subjected to the fracture test described in Experiment 1.

**Figure 4:**
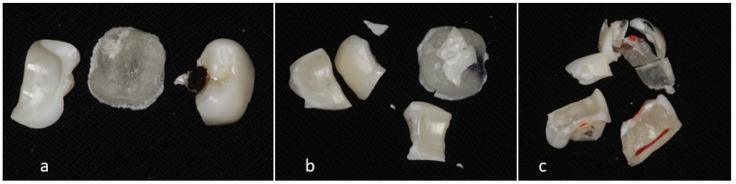
Failure modes: (**a**) crown fracture only, (**b**) crown fracture and core crack, and (**c**) fracture crown and core.

**Figure 5:**
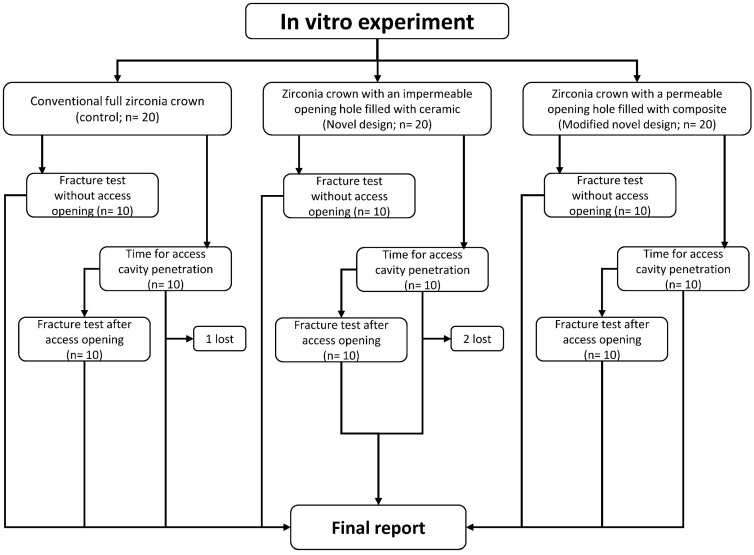
Flowchart of the experiment.

For analysis, the following variables were evaluated: (a) compressive strength of the three different groups before and after access opening; (b) time required for penetration through the different types of crowns; and (c) failure mode of the three groups before and after access opening. For the failure mode, fragments of the fractured crowns were collected, and the fracture patterns were analyzed. Accordingly, the fracture modes were categorized into three groups: (1) crown fracture only, (2) crown fracture with core cracking, and (3) crown and core fracture (Figure 4). The flowchart of the entire experiment is shown in Figure 5.

### Statistical Analysis

The collected data were checked, coded, and transferred for analysis. The results of the normality test revealed a normal distribution of the data (Shapiro–Wilk test; *p* > 0.05). Consequently, parametric tests were employed. Differences in compressive strength before and after penetration were analyzed using an independent *t*-test, as the samples for before and after were not the same. One-way analysis of variance (ANOVA) was utilized to assess differences in compressive strength and the time for initial penetration among the different crown groups. If significant, post hoc pairwise comparisons with Bonferroni’s correction were performed. The Chi-squared test was used to evaluate differences in failure modes. Statistical analysis was conducted using the Statistical Package for Social Sciences software (SPSS v25, IBM Inc., Chicago, IL, USA), with the level of significance set at *p* < 0.05.

## 3. Results

Table 1 shows the mean values of compressive strength for the different types of crowns before and after initial penetration, as well as the time required for initial penetration. The highest compressive strength before initial penetration was recorded for zirconia porcelain crowns (760.2 ± 25.2 MPa), while the lowest was for zirconia composite crowns (652.4 ± 25.9 MPa). However, zirconia composite crowns exhibited the highest mean compressive strength after initial penetration (754.3 ± 177.1 MPa), with a minimum compressive strength value of 491.6 MPa. In contrast, after initial penetration full zirconia crowns recorded the lowest mean compressive strength (478.7 ± 98.2 MPa) and a minimum compressive strength of 264.2 MPa. The longest time required for initial penetration was noted for zirconia porcelain crowns (19.2 ± 13.8 s), while the shortest time was for zirconia composite crowns (2.5 ± 0.8 s). More details are presented in Table 1.

Table 2 shows that the differences in compressive strength before initial penetration were significant for all pairwise comparisons. The greatest difference was observed between zirconia porcelain and zirconia composite crowns (mean difference = 107.8 MPa; 95% CI = 81.3 to 134.4 MPa), while the smallest difference was between full zirconia and zirconia composite crowns (mean difference = 32.1 MPa; 95% CI = 5.6 to 58.7 MPa). After initial penetration, significant differences in compressive strength were noted between full zirconia and zirconia composite crowns (mean difference = −275.6 MPa; 95% CI = −445.0 to −106.3 MPa) and between zirconia porcelain and full zirconia crowns (mean difference = 189.8 MPa; 95% CI = 20.5 to 359.2 MPa). The difference in time required for initial penetration was not significant between zirconia porcelain and full zirconia crowns (mean difference = 6.6 MPa; 95% CI = −3.0 to 16.1 MPa). However, significant differences were observed with the other types of crowns.

As presented in Table 3, the difference in failure modes among the crowns was significant before initial penetration (*p* < 0.001). All zirconia composite crowns exhibited crown fractures and core cracking, while all full zirconia crowns displayed only crown fractures. However, the differences in failure modes between the crowns after initial penetration were not significant (*p* = 0.464).

Difference in failure mode before and after initial perforation was significant only for zirconia composite crowns (*p*< 0.001), with a higher mean value before initial penetration (Table 4). Differences in failure modes before and after initial penetration for the same type of crown were significant only for zirconia composite crowns (*p* = 0.001).

## 4. Discussion

The present study aimed to evaluate the fracture resistance and the time of initial penetration of a novel design zirconia crown and its modification compared to the conventional design, as well as to assess the failure mode of fractured crowns. The null hypotheses were rejected for all variables: fracture resistance, time of initial penetration, and mode of failure. The findings revealed a significant difference in fracture resistance before initial penetration, favoring zirconia porcelain, while the zirconia composite crown recorded the lowest value. However, the result of the fracture resistance value of the zirconia composite crown is premising to tolerate the functional occlusal compared to the full zirconia crowns [9,13,14].

The lower value for the zirconia composite crowns before initial penetration can be attributed to the weaker resistance of the composite, which is located directly under the applied force (the pressure bar). This positioning leads to load transfer to the core, resulting in fractures of both the crown and the core. Conversely, the zirconia composite crowns exhibited the highest value after initial penetration (after penetrating through the composite). This can be explained by the fact that the initial penetration through conventional zirconia and zirconia ceramic may create cracks in the zirconia core, weakening the crown during the fracture test [15]. In contrast, during penetration through the zirconia composite, the core of the crown remained untouched, and all penetration occurred through the composite. As a result, the crowns’ cores remained intact, allowing the samples to behave as if they were new crowns when subjected to the fracture test.

The high compressive strength of zirconia porcelain crowns before initial penetration is consistent with prior research indicating zirconia’s superior mechanical properties due to its high density and fracture toughness [10,11,12]. Conversely, the lower compressive strength of zirconia composite crowns aligns with findings that composites generally exhibit lower mechanical strength compared to porcelain [16,17]. The significant difference in compressive strength before initial penetration between zirconia porcelain and zirconia composite crowns emphasizes the superior mechanical properties of porcelain over composite materials. Additionally, the smaller difference in strength between full zirconia and zirconia composite crowns is consistent with previous studies suggesting that full zirconia and composite crowns can have comparable strength in certain properties [18,19].

Regarding penetration time, it is not surprising that accessing zirconia composite was significantly faster than in the other groups. The negligible difference in penetration time between zirconia porcelain and full zirconia crowns might be attributed to the fact that both types of crowns have similar density and hardness properties, contributing to comparable resistance to penetration [20]. The significant difference in failure modes before initial penetration is consistent with previous studies indicating that composite materials tend to exhibit more complex failure patterns (crown fracture and core cracking) due to their layered structure and lower fracture toughness compared to monolithic materials like full zirconia [21]. The similar failure modes observed after initial penetration and the significant difference in compressive strength for full zirconia crowns support the finding that initial structural integrity can greatly influence the mechanical behavior of dental materials under stress [20].

The significant drop in compressive strength of full zirconia crowns after initial penetration contrasts with studies that highlight zirconia’s durability under stress [21]. This discrepancy may be attributed to differences in experimental conditions or crown fabrication methods. Variations in lab tests versus real-life conditions can cause materials to behave differently. Furthermore, different ways of making the crowns (e.g., sintering temperatures, milling precision, or final glazing techniques) can impact the material’s structural integrity. The substantial decrease in compressive strength between full zirconia and zirconia composite crowns after initial penetration contradicts findings from other studies that emphasize zirconia’s resilience under stress [11,22]. This may suggest potential flaws or weaknesses in the specific full zirconia crowns used in this study. Additionally, the absence of significant differences in failure modes among all groups after initial penetration suggests that initial structural differences may lessen under continued stress, contrary to some studies that report persistent material-specific failure patterns [23].

The significant reduction in the compressive strength of full zirconia crowns after penetration highlights potential weaknesses that could compromise their long-term performance in clinical settings when root canal treatment is performed through such crowns. The notable difference in the failure mode of zirconia composite crowns before and after initial penetration suggests that initial damage can exacerbate underlying structural vulnerabilities, leading to more complex failure patterns. However, in clinical settings, composite bonded to dentine can withstand occlusal forces, and such failures might not be of clinical significance [24]. These findings can be explained through the principles of material science, particularly focusing on the microstructural behavior of dental materials under stress. The significant differences in performance before and after penetration highlight the critical role of internal composition and bonding in determining the strength and durability of these materials, especially regarding fracture mechanics and stress distribution in ceramics and composites. The marked decrease in compressive strength of full zirconia crowns following penetration suggests the occurrence of crack propagation or microstructural damage, which substantially weakens the material [15]. Zirconia-based materials are generally characterized by high hardness due to their dense crystalline structure, leading to similar penetration resistance for both zirconia porcelain and full zirconia crowns [25]. In contrast, composite crowns, with their layered construction, are more susceptible to complex damage (e.g., fractures and core cracking) because stress is distributed across different material layers. Full zirconia crowns, being monolithic, exhibit simpler failure patterns (such as crown fractures), but may experience significant strength reduction after initial penetration due to crack propagation [26].

The unexpected substantial decrease in compressive strength of full zirconia crowns after initial penetration suggests potential microstructural flaws or crack propagation that could weaken the material [15]. Further microscopic and compositional analysis is necessary to understand this anomaly. This significant drop indicates a need to investigate specific causes, such as material defects, manufacturing inconsistencies, or inherent structural properties. The anticipated similarity in penetration times between zirconia porcelain and full zirconia crowns, due to comparable material properties, contrasts with the significant differences observed in other crown types, warranting further exploration of variations in composite structure or fabrication techniques. Additionally, the lack of significant differences in failure modes after initial penetration implies that all crown types may exhibit similar failure patterns under continuous stress, emphasizing the need for more research into the specific stress response mechanisms of different crown materials.

This study’s test conditions may not accurately reflect the real-life performance of crowns, as factors like temperature changes and varying loads are significant in actual use. Additionally, the results may not apply to all zirconia or composite crowns due to differences in material quality and manufacturing processes. The experimental setup may not replicate the complexities of the oral environment, where multiple variables affect crown performance. Variability in material batches can also impact the generalizability of the findings. While zirconia crowns exhibit high initial compressive strength, indicating their durability, the reduced strength after initial penetration suggests caution for patients with high occlusal forces or those prone to bruxism.

The current study has several limitations, including the choice of tooth type, access cavity design, and the materials used for the crown before fracture testing. Moreover, the mechanical loading before fracture test was not performed. The fracture resistance tests may not apply to all scenarios, as different teeth may experience varying occlusal forces, and the placement of holes in the crowns may not always align with these forces. However, the study offers insights into worst-case scenarios. Additionally, due to the variability in endodontic access cavity designs for different teeth, a round design was selected based on options available in the software’s digital library.

The present and previous researches [8] focus on addressing the challenge of creating access cavities through existing fixed prostheses, including crowns on any tooth in the jaw. The goal is to develop an endo-friendly crown that maintains strength before and after root canal treatment (RCT), benefiting both the dentist placing the crown and the one performing future RCTs by reducing concerns about crown damage and facilitating access to the pulp chamber without harming the prosthesis or tooth. The modified zirconia composite crown features a design where only the composite material is penetrated during access, minimizing the risk of cracks in the zirconia compared to full zirconia crowns. After RCT, the tooth can be restored with the same composite material. However, the initial design included an impermeable hole filled with ceramic, which ensured crown strength but complicated access, similar to conventional zirconia crowns. Further studies are recommended to explore filling the hole with composite instead to facilitate penetration and reduce the risk of failure.

## 5. Conclusions

The zirconia-composite crown is an excellent choice for placing new prostheses, as it provides easy access and a predictable guide to the root canal system. Additionally, it demonstrates good fracture resistance both before and after performing root canal treatment.

## Figures and Tables

**Table 1:** Descriptive statistics of compressive strength (MPa) and time (seconds) of access opening for the different types of crowns.

Type of Crown	N	Mean	SD	Minimum	Maximum
Compressive strength before initial penetration					
Full Zirconia	10	684.5	17.8	657.4	710.4
Zirconia Porcelain	10	760.2	25.2	726.3	804.2
Zirconia Composite	10	652.4	25.9	620.7	703.9
Compressive strength after initial penetration					
Full Zirconia	10	478.7	98.2	264.2	611.3
Zirconia Porcelain	10	668.5	158.2	423.4	881.5
Zirconia Composite	10	754.3	177.1	491.6	1067.1
Time required for initial penetration					
Full Zirconia	9	12.6	2.6	10.3	18.0
Zirconia Porcelain	8	19.2	13.8	8.0	48.4
Zirconia Composite	10	2.5	0.8	1.5	4.0

**Table 2:** Differences in compressive strength (MPa) and time (seconds) between the different types of crowns.

Type of Crown		Mean Difference	95% CI of the Difference		*p*
			Lower	Upper	
Differences in compressive strength before initial penetration					
Full Zirconia	Zirconia Composite	32.1	5.6	58.7	0.014
Zirconia Porcelain	Full Zirconia	75.7	49.2	102.2	0.000
	Zirconia Composite	107.8	81.3	134.4	0.000
Differences in compressive strength after initial penetration					
Full Zirconia	Zirconia Composite	−275.6	−445.0	−106.3	0.001
Zirconia Porcelain	Full Zirconia	189.8	20.5	359.2	0.024
	Zirconia Composite	−85.8	−255.1	83.5	0.621
Differences in time of initial penetration					
Full Zirconia	Zirconia Composite	10.1	1.1	19.1	0.024
Zirconia Porcelain	Full Zirconia	6.6	−3.0	16.1	0.266
	Zirconia Composite	16.7	7.4	26.0	0.000

**Table 3:** Differences in failure mode between the different types of crowns.

Type of Crown	Failure Mode			*p*
	Crown Fracture Only	Crown Fracture and Core Cracked	Crown and Core Fracture	
Differences in failure mode before initial penetration				
Full Zirconia	10 (100.0)	0 (0.0)	0 (0.0)	0.000
Zirconia Porcelain	9 (90.0)	0 (0.0)	1 (10.0)	
Zirconia Composite	0 (0.0)	10 (100.0)	0 (0.0)	
Total	19 (63.3)	10 (33.3)	1 (3.3)	
Differences in failure mode after initial penetration				
Full Zirconia	8 (80.0)	1 (10.0)	1 (10.0)	0.469
Zirconia Porcelain	10 (100.0)	0 (0.0)	0 (0.0)	
Zirconia Composite	7 (70.0)	2 (20.0)	1 (10.0)	
Total	25 (83.3)	3 (10.0)	2 (6.7)	

**Table 4:** Differences in compressive strength (MPa) within groups before and after initial penetration.

Type of Crown		Mean ± SD	Mean Difference	95% CI of the Difference		*p*
				Lower	Upper	
Full Zirconia	Before	684.5 ± 17.8	205.82	135.06	276.58	0.000
	After	478.7 ± 98.2				
Zirconia Porcelain	Before	760.2 ± 25.2	91.68	−22.06	205.43	0.102
	After	668.5 ± 158.2				
Zirconia Composite	Before	652.4 ± 25.9	−101.96	−229.16	25.25	0.104
	After	754.3 ± 177.1				

## Data Availability

Data available on request from the corresponding author upon a reasonable request.
